# What's emotion got to do with it? Reflections on the buildings of the Portuguese (Family) Courts

**DOI:** 10.3389/fsoc.2024.1412161

**Published:** 2024-10-14

**Authors:** Patrícia Branco

**Affiliations:** Centre for Social Studies, University of Coimbra (CES-UC), Coimbra, Portugal

**Keywords:** courthouse buildings, emotions, Lefebvre's lived space, court users, Portugal

## Abstract

Courthouses, as public edifices, serve as the physical backdrop for the administration of justice. Simultaneously, they are spaces inhabited and visited by a diverse array of court users, ranging from judicial professionals to litigants. This article explores the nuanced interplay between courthouse spaces and the emotional experiences they generate. It starts by surveying existing studies that examine such an intricate relationship. Then, and by drawing from a sample of interviews conducted across two distinct time periods (2010-2011 and 2017-2019) in Portugal, the article delves into the lived experiences of judges, prosecutors, and litigants. Their narratives provide a multifaceted view of the emotional experiences associated with the Portuguese (Family) Court buildings. To analyse these experiences, I turn to Henri Lefebvre's concept of lived space. Lived space refers to the emotions, memories, and interactions within a particular spatial context. Such dimension, in relation to courthouses, directly connects to the lived experience of legitimacy loss and low self-esteem affecting decision-making, on the one hand, and estrangement and rights' exclusion, on the other hand, felt by those subjects. By investigating how the spatial configurations of courthouses shape our emotions, we gain insights into the profound impact of such built environments on our understanding of the justice system, and the physical and symbolic obstacles in accessing it.

## 1 Introduction

(…) the tears and the turmoil of family strife characterize family court. We know they are law-related tears when they are shed in and around the court.

John Brigham, *Seeing Jurisdiction: Some Jurisprudential Issues Arising from Law Being “... All Over”* (2009, p. 386)

Architecture, insofar as it is linked both to the outside world and to society, through the relationships established between social framework, culture, and technique, incorporates and creates the contexts in which feeling is produced. Emotions are thus embedded in particular contexts (Roach Anleu et al., 2016). Such a particular context, or setting, is the courthouse building. For Dahlberg (2009), in a courthouse there are strong emotions at play, which are shaped and co-created by the physical design of the space and the expression of seriousness of the professionals involved (along with procedural rules and the rationality of the law).

Courthouse edifices consequently can prompt a variety of emotional responses. These spaces have the potential to evoke positive and negative feelings, contingent upon the circumstances and users involved. Therefore, the expectations and experiences expressed by users regarding these buildings may vary significantly. Contrary to the notion of these spaces as neutral settings, devoid of influence on their occupants, court buildings play an active role in shaping societal perceptions of justice. Far from being mere physical structures, they impact the interpretation and experience of justice. Their design influences the overall experience of justice, or what Schliehe and Jeffrey (2022, p. 2) call the ‘lived experience of the justice journeys'.

How then do court buildings shape emotions and perceptions of justice for different users, such as judges, prosecutors, and parties?

To analyse their emotional experiences, I turn to Henri Lefebvre's concept of the lived space. In his seminal work, *The Production of Space* (Lefebvre, 1991[1974]), Lefebvre asserts that space is not a mere container or neutral framework; rather, it is a dynamic social product. My idea of working with Lefebvre's theoretical framework stems from Dahlberg's (2009) notion of the courtroom as a special kind of social space and from Schliehe and Jeffrey (2022) concept of the lived experience. Lefebvre discerns three dimensions—perceived space, conceived space, and lived space. Such dimensions are thoroughly present in court edifices and shape the perspectives of judicial professionals and litigants in regard to the justice system. I focus on the dimension of the lived space, which offers a lens to examine the multiple ways the spaces of the courthouse are experienced, and the emotions it evokes.

In the subsequent section, I undertake a comprehensive review of the extant literature that investigates the interconnections between courthouse architecture and emotional responses. Following this, in Section 3, I scrutinize Henri Lefebvre's triadic conceptualization of space—conceived, perceived, and lived—in the context of courthouse environments. Section 4 delineates my methodological framework, with a particular emphasis on the application of thematic analysis.

In Section 5, drawing upon a dataset of interviews conducted during two distinct periods (2010–2011 and 2017–2019) in Portugal, the article explores the lived experiences of judges, prosecutors, and litigants, categorized as user-inhabitants and user-visitors. This section examines the lived space from the perspective of inhabitants, focusing on their experiences of perceived threats to objectivity and quality in judicial decision-making and sentencing, as well as their experiences of empathy. Additionally, it investigates the lived space from the perspective of visitors, highlighting their experiences of estrangement, distress, and exclusion of rights. Through this analytical lens, the study elucidates the significant impact that the architectural design of courthouses exerts on our comprehension of the justice system, as well as the physical and symbolic barriers that impede access to justice.

## 2 Courthouses' spaces and emotions: exploring the existing literature

The literature surveying court users' experiences and feelings connected to the courthouse buildings is somewhat limited, but very insightful. While some authors have used ethnographic work inside the courthouse, observing hearings and photographing courtrooms (Ouviña, 2014; Perrault, 2020), and others have conducted research with undergraduate (law and psychology) students using photos (Maass et al., 2000; Clinton and Devlin, 2011; Chase and Thong, 2012), the most interesting research explored such subjective experiences through interviews with defendants (Schliehe and Jeffrey, 2022), crime victims (Toews, 2018), and asylum appellants (Gill et al., 2021). The present article also explores interview material (I will deal with this in more detail in Section 4).

Kafka's *The Trial* is perhaps the most illuminating example of a novel illustrating the role of court buildings in shaping public perceptions of law (Jeffrey, 2019). K. is forced to explore the dark spaces of the court, rambling on in a careless, almost morbid, atmosphere, facing stairs and floors that look like an Escher drawing, left to himself, without any point of orientation in that legal labyrinth, which leaves him with feelings of emptiness, oppression, and precariousness (Nitrato Izzo, 2013).

Architectural features and façades of courthouses (and police stations) are thus said to influence how users perceive authority, professionalism, and legitimacy, or the lack of it, of the justice system (Clinton and Devlin, 2011). Likewise, courtroom settings, and judicial attire, are said to affect the evaluation of judicial behavior, as the judge in robe is seen as more respectful because the black robes and the marble columns are associated with a sense of authoritative and unbiased justice (Chase and Thong, 2012). As suggested by these authors, it is therefore possible to assume that participants—especially if they are not repeat players—will feel disrespected and disinclined to trust the judge if the court in which they are heard does not live up to expectations—as happened with K.

In her study of the Donostia-San Sebastian penal courthouses, Ouviña (2014) argued instead that the solemnity of the buildings, courtroom, and robes, are aspects litigants, and victims in particular, are not familiar with, which can generate a feeling of distance about justice. Toews (2018) research also points to participants frequently referring to the court buildings as cold, hard, and distant. After conducting semi-structured interviews and focus groups with survivors of violence and representatives of community organizations, Toews' findings revealed that crime victims associated courthouse architecture feelings of insignificance, unwelcoming, inhumanity, and a high potential for revictimization.

Issues of discomfort and stress have also been correlated with intimidating design by Maass and colleagues. In their study, they compared two courthouses, with completely different styles, located in Padova (Northern Italy): the old one located in a former convent, and the new courthouse, built in 1991 and in use since 1995, situated in a modern building. Participants imagining themselves accompanying a friend to the courthouse experienced greater discomfort and stress when facing a trial in the modern courthouse than in the older one, associating such feelings with an increased probability of being convicted (Maass et al., 2000). A recent study conducted by Song and Zhao (2023) amplified this question in terms of the audio impact inside the courthouse, having investigated the influence of the sound environment at court on the defendant's emotions.

There is thus an intriguing point to note here: court buildings project a sense of majesty and solemnity, they command respect and project an image of unbiased justice, which can be perceived positively. At the same time, solemnity can be associated with disrespect and intimidation. Conversely, if courthouse buildings appear too modern or mundane, they may look less trustful, but this could potentially foster a greater sense of equality. This ambivalence presents an interesting conundrum. The balance between maintaining respect and trust, while ensuring fairness, is therefore delicate and complex.

Jandura (2018), on his part, claims that certain physical elements in the courthouse design—like crowded corridors or waiting areas, the absence of natural light, or lack of legible wayfinding, to which we can add security barriers to entry or the (in)availability of refreshment (Schliehe and Jeffrey, 2022), features which are different from the issue of solemnity/mundanity—can also trigger negative emotions. Schliehe and Jeffrey (2022), drawing on interviews with 455 defendants who were convicted in criminal courts in England and Wales, examined how defendants perceive trial spaces and how such perceptions shaped their experiences of justice: their narratives conveyed feelings of unfairness, missing respect, a sense of being silenced, which led to defendants doubting the existence of justice. For such reasons, Toews (2018) argues that design should provide psychological relief, privacy, and safety.

Gill et al. (2021) came to similar conclusions. After observing asylum appeals in the U.K., and interviewing former asylum appellants and legal representatives, they identified disorientation, distrust, and disrespect as qualitative obstacles to access to justice. When appellants were confronted with the (often difficult to find) location of hearing centers they frequently experienced disorientation; when they entered the deceptive and cold atmosphere^1^ of the tribunal they felt intimidated or disrespected, and their participation and engagement in the hearing was severed. All these aspects resulted in a perception of unfairness and as a threat to access to justice.

Moving away from the penal context, Perrault (2020) examined the Chambre de la Jeunesse^2^ in Montreal, Quebec, in Canada. The issue of privacy, or the lack of it, was one of the dimensions the author analyzed. Complaints concerned discomfort and privacy, especially regarding corridor furniture and the private nature of the procedures. Meetings between lawyers and their clients took place in the few partitioned offices provided, meaning that several discussions, of a confidential nature, were held in the corridors, without the possibility for the people involved sitting down. This led families, parents, young people, and victims to feel uncomfortable and concerned with the possible echoes of their conversations likely to reach the public waiting outside.

As said previously, it is important to examine the literature exploring users' experiences with the architecture and design of justice buildings. By narrating their emotional experiences in court spaces we can infer that positive experiences might foster trust and confidence, while negative experiences (dealing with discomfort, lack of privacy, disorientation, intimidating design) might lead to distrust or deception, which will influence how users interact with the justice system. Positive and negative experiences can serve to inform reforms of the justice system and replicate best practices.

The literature review provided here does not aim to be exhaustive, nor could it be, as it mainly considered texts in English and French, thereby excluding other contexts. My objective was to identify architectural and organizational aspects of the court spaces that directly influenced the emotions and experiences of participants, thereby emphasizing the significance of considering building designs' impact on individuals' interactions with the justice system. Furthermore, my intention, with the present article, is to extend beyond criminal court studies, which make for the bulk of the existent literature, and to incorporate research on family courts, thus adding another contribution to it. Finally, the studies examined have not dealt with the professionals' emotions, and so this article covers that gap, not only by exploring the lived spaces of judges and prosecutors (the inhabitant-users), but also because that analysis is important as it relates emotional responses to space and its potential effects on sentencing and legal decision-making. As for the litigants and witnesses (the visitor-users), it moves beyond Perrault study (2020) of the Chambre de la Jeunesse, not only because Portuguese Family Courts have a broader material competence, but because my analysis incorporates interview data, capturing the specific details of experiences.^3^

## 3 Lefebvre's lens on the court building as a lived space

Lefebvre argues that space is socially constructed, reflecting power relations, ideologies, and everyday practices. Space can thus be characterized as a triad of spatial practices, representations of space, and spaces of representation. This triad alternates with another one, that of the perceived, of the conceived, and of the lived space. Thus, spatial practices produce perceived spaces, representations of space relate to conceived spaces, and spaces of representation are assessed as lived spaces (Lefebvre, 1991[1974]; Stanek, 2007; Leary-Owhin, 2015).

Perceived space is the physical organization of space, such as the buildings, streets, and infrastructure that shape the daily routines and activities of people. Conceived space is the space created and imagined by urban planners, architects, and other professionals, who impose their visions and ideologies on the spatial layout. Lived space is the individual, subjective experience of space, shaped by personal emotions, practices, and symbols: hence the space of ‘inhabitants' and “visitors” (Lefebvre, 1991[1974]; Lampropoulos et al., 2020). The notion of lived space is one of Lefebvre's central contributions, as it refers directly to bodily lived experience (Lumsden, 2004).

Court buildings can thus be seen as an illustration of conceived, perceived and lived space. They are conceived spaces for they are designed and built by professionals (architects, technocrats, and political decision-makers, linked to the ministries of justice) who have a certain idea of what justice and judicial authority should look like. By using specific architectural elements, they create a symbolic and ritualistic spatial setting that conveys, and imposes, the power and legitimacy of this institution and of the legal profession. The perceived space of the courthouse is the physical layout and organization of the (court)rooms, entrances/exits, corridors, furniture, equipment. It is linked to the way they are seen and used. Finally, the lived space of the court buildings is the emotional and subjective space that is experienced and imagined by those who participate in the courtroom dramas (Dahlberg, 2009), i.e., the diverse types of court users (judges, prosecutors, lawyers, litigants, witnesses, court staff, and even the public). These users may experience different feelings and emotions regarding the buildings and internal spaces, depending on their roles, status, (cultural and emotional^4^) backgrounds, and (legal) expectations.

The dimension of the lived space hence brings our focus back to the complex meanings that subjects create in and with space (Michon, 2024). Lived space provides the context for emotional encounters, affecting our well-being, stress levels, and social interactions. Lefebvre's concept thus enriches our understanding of the role courthouse spaces play by emphasizing the lived experience, the individual emotions. I am aware that Lefebvre's work has been adapted for various uses by socio-legal and critical legal scholars, particularly regarding the produced nature of space or the concept of the right to the city (Butler, 2018). In this article I am not engaging in the discussion on the nature of space in relation to its normative framework. I am interested in Lefebvre's dimension of the lived space, which highlights how space comes to have particular meanings for an individual, encouraging us to explore these layers of meaning and experience, and the lessons we can learn from it. Such a lens helps us to gain a better grasp of the importance of understanding the subjective experiences within spatial contexts by directly relating space to emotions, and so to the production of meaning which is connected to particular spaces. In addition, it introduces a layer of theoretical sophistication to studies on the justice system, courthouse architecture and access to justice.

## 4 Methods and data

The analysis I present in this article builds on data from two research projects I coordinated in two different periods: 2010–2011 and 2017–2019. The research question which guided my research on courthouse architecture was informed by this idea: the importance and relevance of the courts' physical spaces and everyday practices to research on access to justice. I chose the Family Courts as my case study because family justice addresses situations of great social conflict, emotional fragility, and personal vulnerabilities. In Family Courts emotions are clearly palpable, as the people involved are often suffering from very painful legal and psychological conflicts (Vasconcelos, 2010)—the tears that are shed in and around the court (Brigham, 2009). For many families, the interaction with the judicial system is associated with overwhelming feelings and numerous emotional issues, such as the tension divorce implies (many times involving domestic violence); highly conflictual cases involving parental responsibilities; juvenile delinquency; and neglected children—the type of cases that fall under the material competence of Family Courts in Portugal (see article 122, Law no. 62/2013, from August 26th). As Dahlberg claims, these are a “very emotionally charged kind of private case” (2009, p. 185).

Family Courts, given their material competence, are different from the criminal courts (which are more commonly examined) and need to be analyzed apart from the criminal court model. The emotion's perspective, however, was not something I had pondered, but it was there, naturally. I understood it at a later stage. This article is thus an exploration of the nuanced interplay between courthouse buildings (in Portugal), emotional experiences, and perceptions of (un)access to justice. For a detailed account of the methodological outlines of my research, see Branco (2023).

In this article, as I said, I will examine the emotionality lived inside the Portuguese Family Courts buildings vis-a-vis the lived space experiences of the diverse users. To do so, I will rely on the interviews I conducted with user-inhabitants, such as judges and prosecutors,^5^ and with user-visitors (litigants).^6^ How space comes to have particular meanings for an individual can only be expressed by that individual alone (Michon, 2024). Interviews, therefore, play a crucial role in understanding lived space, for they allow the researcher to delve into users' (inhabitants and visitors) experiences and emotions within the courthouse walls. The interviews are the most organically capable method for capturing the context-specific details of experiences, which might be missed by other methods (such as ethnography, for example), allowing hearing, encouraging speech. This process thus permits access to the lived space, the most elusive of Lefebvre's dimensions (Michon, 2024) because of its subjectivity.

Between October of 2010 and October of 2011, I conducted a total of 27 interviews (six judges and four prosecutors working in Family Courts, and six with litigants/witnesses; the remaining interviews were conducted with attorneys, architects, and relevant decision-makers) (Branco, 2018). In the period 2017–2019 I conducted 17 interviews with 14 key stakeholders (three judges and two prosecutors; the remaining interviews were conducted with attorneys, court officials, mayors, and representatives of the Ministry of Justice) (Branco, 2019). The semi-structured interviews, ranging from 30 to 120 min in duration, were audio-recorded and subsequently transcribed verbatim manually, without the aid of speech-to-text converter software. All interviews were conducted in Portuguese and later translated by the researcher. Anonymisation was carried out in compliance with the research ethics code of conduct. In the selected quotations, participants are identified by their roles followed by a sequential number and the period during which the interview took place.

A thematic analysis was performed on the interview data, enabling the authors to identify, examine, and interpret recurring patterns of meaning within the qualitative data collected (Braun and Clarke, 2006). This method facilitates the construction of common variables to analyze how individuals refer to the same topic—such as courthouse buildings, experiences, and feelings—either similarly or differently (Schwarze, 2023). Consequently, thematic analysis proves to be an effective approach for uncovering individuals' views, opinions, and experiences from a qualitative dataset. The data was coded by highlighting sections of the transcribed interviews or individual sentences, and assigning shorthand labels or “codes” to describe their content. This codes turned into themes, giving form to Sections 5.1 to 5.5.

Interviews with judges and prosecutors, in addition to being important to understand the lived space of these user-inhabitants, also serve to deepen the issue of emotions and legal professions, deeply related to objectivity and quality in decision-making and sentencing, bringing to the fore the connection with space. While the interviews conducted with user-visitors are fewer than those conducted with the user-inhabitants (the professionals), the insights gathered significantly resonate with and strengthen the existent international comparative panorama through the lens of the Portuguese context.

## 5 Courthouse narratives: the lived spaces of inhabitants and visitors in the Portuguese courthouse buildings

Portuguese courthouses have multiple and/or varied architectural profiles, which can be classified in terms of the coexistence of different architectural styles from different (political and temporal) periods. Thus, we find buildings, inherited from the dictatorship period (which lasted from 1926 to 1974), that are monumental in scale and present grand entrances, columns and profusely decorated façades and courtrooms. At the same time, the buildings constructed or adapted during the democratic period (from 1974 onwards) exhibit an architectural model which can be characterized as heterogeneous, alternating columns with apartment-like layouts and banal décors.

Concomitantly, in any report dealing with the state of maintenance in the (Portuguese) courts, we are presented with images of courts operating in buildings in poor conditions or where parts of the building threaten to collapse; in buildings where the temperature rises, due to the lack of air conditioning, leading to hearings being suspended; in buildings where the rain comes in; courts where users have no waiting rooms, where the toilets are out of order or where the elevators constantly break down. We hear of courts where there aren't enough courtrooms to carry out the various types of hearings, or where judges must share tiny offices and carry out hearings in their own offices because of the lack of courtrooms. We also know that not all courts have metal detection gates or, if they do, they are often out of order or broken. Courts where the electricity grid is down, where the computers are old and slow. Situations are often reported, but little is done by the responsible institutional bodies.^7^

Such characterization presents the combination of the conceived and perceived spaces of Portuguese courthouse buildings. In the next subsections I present the lived spaces of judges, prosecutors, and litigants/witnesses. Their narratives provide the complex meanings that these subjects create in and with such particular spatial settings.

### 5.1 Lived space as the inhabitants' experience of legitimacy lost

The interviewed judges and prosecutors narrated a complex interplay of expectations and feelings. They experience exhaustion, frustration, a sense of loss of legitimacy when the physical conditions fail to match the importance not only of their professional roles but also of the judicial institution.

*I find this building to be absolutely unqualified. What will people think of this? Will they think this is a courthouse? I have had litigants here who've asked me “And now when are we going to court?”!* [Judge 1, 2011]

*This continuity of immense corridors, an all-white corridor, with a gray linoleum on the floor, is absolutely depressing. And in a court of law, it doesn't lend it much dignity. This issue of dignity may seem a false question, but it is an important one. Because it is not, obviously, due to the dignity of the materials that the exercise of the function is dignified. But, for a person who rarely goes to court and enters a building that, externally and internally, looks the same as all other office buildings, but perhaps even with less quality, with less appearance… And, moreover, if the hearing takes place in the judge's office, if you don't even go to the courtroom, which always has some distinction in terms of space, you won't even realize that you are in a courthouse!* [Judge 4, 2011]

The quoted excerpts are a clear illustration of this sense of loss of authority, of unaccomplished expectations that relate to a building without any dignity, quality, or distinction to serve its function. Inadequate resources, outdated facilities, and absence of proper conditions exacerbate this frustration and raise questions on the symbolism and power of the judiciary. The question “Will they think this is a courthouse?” (echoing Clinton and Devlin's study on police stations), accentuates a feeling of disenchantment toward the institution, amplified by the constatation that the courthouse becomes an administrative building, and the hearing lacks ceremony (Lucien, 2010). Furthermore, the next observation suggests a potential intentional strategy to promote professional efficiency at the cost of symbolism:

*It's all white, it looks like a hospital. I think that, more and more, courts look like hospitals. Maybe it's on purpose, so we don't get too distracted*. [Judge 3, 2011]

The professionals also acknowledge the changes that occurred regarding the conceived space of the buildings in terms of architectural styles. They express, nonetheless, a sentiment of overstretched lines that contribute to a discredit of the judiciary when the buildings suffer from a complete scarceness of those characteristics that conceptually embody the courthouse symbolism. They argue that such changes have transformed the foundational conceived and perceived spaces of the courthouses, challenging the configuration and symbolism of the lived space in which they found their function. At the same time, they understand the courthouse as a space of dignity, that should match the expectations of all users by being comfortable, welcoming, and accessible.

*I think the architecture of the courts has become a little desacralized and thank goodness for that! We no longer have the idea that the court must be upstairs for people to “ascend to heaven”. We now have some courts that are much closer and accessible to people. However, and to a certain extent, I believe the extreme happened, when the courthouse got mixed with [office] buildings. I think there it also loses. The symbolic function, which is important, is lost. And when people go to court, they also look for the symbolism of the court. This symbolism is also in the building. It doesn't have to be an imposing building, it doesn't have to be a building that scares people, as was the idea [before]. I think it must be a functional building, but it must be a building with dignity, where people feel welcomed, where they feel comfortable. With working conditions too, where people work in good conditions*. [Prosecutor 3, 2017]

### 5.2 Lived space as the inhabitants' experience of threat to objectivity and quality in decision-making and sentencing

As I described before, Portuguese court buildings present a series of maintenance problems. Perceived space translates into a lack of working and safety conditions, which have to do with uncomfortable buildings (absence of air conditioning, natural light, and adequate furniture), inadequate use of space (insufficient spaces to work comfortably), and outdated infrastructure. These conditions influence the lived space of the inhabitants: the creaking chairs, the piles of files, the peeling paint, mirror the system's defectiveness. By complaining about their everyday working conditions, feelings such as anger and frustration are repetitively present:

*Working in a place surrounded by files, where the files aren't where they should be, which is on the shelves, but are on the chairs, on the windowsills... No, I can't stand it! There are offices where there isn't even a table to put the files on. (…) where the floor is all dirty, there's no varnish, the carpet has a hole in it and a vase is placed on top. I'm fed up with letting it be! (…)* [Prosecutor 2, 2017]

Moreover, judges and prosecutors reveal their experiences of insecurity and powerlessness, exposing the courthouses' spatial disruptions related to conflict, which magnifies inside the family courts. These narratives of their lived space provide the context for social interactions and emotional encounters affecting their well-being and stress levels, revealing a sense of exposure that should not occur in relation to their role.

*The issue of security: I always pray to Our Lady of Fátima that the courts don't have any problems! We are completely open, exposed*. [Prosecutor 1, 2011]

*There is a general amnesia about the conflict environment that exists in the family courts and the danger that this implies. We don't exactly deal with saints, because everything happens here, from people with mental disorders to people with a criminal record, with personality problems, etc... And complicated situations arise. There should be a clear concern with the protection of those who are serving State's authority, because we are not imposing our authority or using authority for our own personal purpose (…). There should be concern, for example, with chairs and materials that are easily thrown, they should be fixed to the floor. Years ago, at the court in XXX, we all had to run away, the large wooden benches that were in the atrium were all thrown at us, and everything was broken. It was a very complicated situation*. [Judge 1, 2011]

The issue of the direct relationship between the physical conditions of the workplace, in this case the courts, and the levels of stress and low self-esteem of magistrates has been a topic under debate recently, in Portugal (Dias et al., 2024) as in other countries. In November 2018, the Lord Chief Justice presented his annual report to the British Parliament, in which he denounced with concern the low self-esteem of the judiciary and how the state of dilapidation of the courts in England and Wales contributed to this (Judiciary of England and Wales, 2018). He added that it would be completely unreasonable to expect magistrates and court officials to work in such conditions, conditions that would be intolerable in any other activity. Also in England, the UK Judicial Attitude Survey of 2017^8^ revealed that 76% of judges felt their working conditions had deteriorated greatly in the preceding 5 years, and 43% of judges felt that the state of maintenance of their court buildings was poor. In a report written by two professors from the University of Cambridge (Turenne and Bell, 2018) about the attractiveness of the judicial function in the United Kingdom, one can read some excerpts from interviews with English and Welsh lawyers and magistrates, where questions of malaise, self-esteem and sad emotions were evident.

This brings back the questions enunciated by Clinton and Devlin study (2011): when the expectations of professionalism and authority do not correspond, professionals may be seen as unskilled. Furthermore, their decision-making capabilities and legal expertise may be weakened by the “dilapidated state of the buildings”. Lefebvre's spatial theory reminds us that the physical space of a courthouse—its layout, and comfort—shapes judges' experiences and, consequently, we may question if this might affect their rulings. A direct link was actually made between self-esteem and decision-making, being self-esteem “very important for getting things done, for deciding” (Prosecutor 3, 2017).

Jerome Frank and other realists were ridiculed for supposedly having said (which was never proven) that how a judge decides a case depends on what they had for breakfast. American realists were, in fact, associated with the idea of “breakfast jurisprudence”. Frank and other realists never maintained that it all boils down to “what the judge had for breakfast”. However, he would not deny that this could influence the decision (Tumonis, 2012). My reflection goes in the same direction, not in relation to what the judges eat,^9^ but in relation to the settings in which they work—in poorly maintained, run-down buildings, looking like hospitals, sitting on uncomfortable chairs,—and the impacts these might have on objectivity and quality in decision-making and sentencing processes. However, it's important to note these are just potential correlations, and a direct connection between the two might not exist. In any case, it would be crucial to address both aspects to ensure the overall effectiveness and efficiency of the justice system.

Judges and prosecutors, however, seem to show no concern about these issues, not only because they are mindful of their professional status and responsibility, but also because they are part of a professional culture that values strength and self-sacrifice (Roach Anleu et al., 2016). This relates also to the judiciary's working experience: routinization can lead to emotional alienation (Bergman Blix and Minissale, 2022). The next quote confirms this:

*I think magistrates have never said much about this, despite the conditions in which we work, because we are in court to solve the cases that come our way. I, and I think most of my colleagues too, put the issue of comfort and of the decoration of our offices aside, because work absorbs us so much that these things just vanish*. [Judge 2, 2011]

Judge 2 is calling attention to his own lived space made of professional expectations, which relate to those of his colleagues as well. By implying they care little about the issue of comfort and that their only concern is to work on their caseload, Judge 2 is revealing a feeling of resilience which fits with a reliable profession, capable of working hard and of upholding objectivity, integrity and ethical standards despite the spatial disruptions affecting their lived space.

### 5.3 Lived space as the inhabitants' experience of empathy

The magistrates interviewed also agreed that the courts can cause fear and intimidation to the visitors, and they are sensitive to that. As Prosecutor 2 said^10^:

*Even nowadays we still meet a lot of people who say “I've never been to this place”; and people come into the court and are frightened to talk to us. And I've often found myself saying to them: “Look, it's nice to come to court. Don't you like it here? Has anyone treated you badly? It's just nice people here”, to break the ice a bit, because you feel that people are nervous*. [Prosecutor 2, 2011]

In the context of family legal proceedings, professionals acknowledge it is incumbent upon them to ensure that the court environment minimizes discomfort and respects peoples' emotional state, in particular if children are involved.

*I need the child to be comfortable telling me what they have to say and what is painful for them. It is always painful because a child's place is not in a court of law. (…) I often bring some toys from the family home, some drawings, and things like that. To try to create this proximity. One cannot approach a child dressed in a black robe, and inside a courtroom, [a room] completely cold and distant…* [Judge 2, 2011]

This empathy both professionals display, understanding and connecting with others' emotional experiences inside the courthouse, relating to how such space can affect people's interactions and stress levels, is also experienced as essential for just decision-making, which involves continuous work to ease the emotional burden of the proceedings.

### 5.4 Lived space as the visitors' experience of alienation and distress

Lived spaces are imbued with emotional significance, as was mentioned. I now turn to the visitors to examine their emotional responses to courthouse spaces. One of the issues that most affected the litigants' experiences had to do with the recognizability of the building as a courthouse, or the opposite of it. Their lived space reveals a mismatch between the expectation and the perception. As one of the user-visitors said:

*That's hardly a courthouse, that's a house... That's just a building. … it's a normal space, as if it were, I don't know, something else*. [Litigant 6, 2011]

This quote reveals Litigant 6′s frustrated expectations regarding a building seen unfit to stand as a physical embodiment of justice. The symbolic meaning of the courthouse was not respected due to the spatial insignificance of the building, seen as a normal building, as something else, but not as a courthouse.^11^ There is a sense of estrangement toward the (ineffectual) symbolism of the building exteriors (Clinton and Devlin, 2011).

Regarding the interiors, the experiences of the visitor-users in the Portuguese Family Courts were particularly marked by two different spaces: the waiting areas and the hearing room/courtroom. In regard to the latter, it is worth noting that the spaces where the hearings/trials took place left an indelible mark, especially the design of the courtroom, evoking that of the criminal court, and creating the perception of being condemned and under arrest, even though the issue related to divorce matters:

*It seems like we owe everyone money. The judge seems like a crow that appears there to devour someone. I'm sorry for saying this, but that's what I felt! Because it wasn't a crime that was being tried, it was a divorce!* [Litigant 6, 2011]

Litigant 6 further recalls his experience of feeling insignificant:

*I felt small in there, and I was a soldier, I fought war overseas! I don't think I was as scared during the Ultramar war as I were there. It wasn't fear, it was that reverence, that really scary environment. Everyone seems to be imprisoned!* [Litigant 6, 2011]

The justice system seems to always equate with a punitive character and with a “space of sacrifice” (Lucien, 2010, p. 186)—where the judge looks like an evil crow about to devour the litigants. Even if the material competence in question is different, as is the case with family courts and its civil nature. Litigant 6 describes a feeling of liability toward society (owing money to everyone), of scare and fear—the memories of the war were pale in comparison to what being inside the courtroom felt like. His lived space of the courtroom speaks of high levels of stress and estrangement.

While the primary function of the symbolic space where the trial takes place, that is the courtroom, is to legitimize the institution, it nevertheless intimidates and even marginalizes the inexperienced (Perrault, 2020), no matter the layout. The experience of being inside a courtroom can thus be quite intimidating, even if it is an adult we are talking of. While adults may find the experience daunting, it is reasonable to assume that the impact on a child can be significantly more profound. As the next quote tells, Litigant 2 confronts her lived experience with the emotional response she believes her child could feel:

*Even for a child, for example, seeing a court like that, I think the child would leave there more scared than when she arrived. For us, it's scary, for a child, I think, it must be even worse*. [Litigant 2, 2011]

The waiting areas in the courthouses are, also, of considerable importance since this is the space where the parties wait for their cases to be heard. Nevertheless, what court user-visitors narrated during the interviews was mostly a sense of vulnerability, distress, and exposure [in many ways identical to what Gill et al. (2021), Perrault (2020), and Toews (2018) have identified in their studies]. The next quotes highlight lived space as a strong sense of emotional and physical distress:

*There was no privacy, and we were left there in the hallway. Man, we were here, and the other party was there, a meter or so away. I was a little distressed. I felt exposed there, you know? I didn't feel physically threatened, but I felt uncomfortable, and since I couldn't let my friend down, I maintained my pose. But I think there is no concern with separating the parties*. [Litigant 1, 2011]

*In that courthouse, if there were some rooms where we could be a little more reserved, we would have a little more privacy. Especially because we were there talking to our lawyer, and it had to be kept quiet so that we could have as much privacy as possible, since there were a lot more people there*. [Litigant 5, 2011]

Such emotional and physical distress can be high in family courts, especially when it comes to divorce cases or parental responsibilities. Having parties together in the same waiting areas may lead to a tense ambience that will project into the way the trial or hearing will take place.

Users invoke court experiences in other countries to better illustrate how different buildings have impacted their lived experiences. Comparing memories, expectations and experiences conveys a sense of self-awareness and allows relational reflection on emotions, in different contexts. The next quote shows a tension between different spatial practices (those between Portugal and abroad) and how the expectations were matched, giving the user a sense of justice he had not felt before:

*What did I see there [referring to the experience in a Danish courthouse]? I saw a modern building, and there was a witness room, a place for the police. And I realized that when there are several parties, they make sure to safeguard the different parties. [Buildings] With lots of light and lots of space, and in good condition. The waiting room had comfortable sofas. And I waited there calmly. A pleasant space*. [Litigant 1, 2011]

### 5.5 Lived space as the visitors' experience of rights' exclusion

The users' experiences also depend on their ability to participate in the processes. Participation is not just a matter of understanding technical language and verbalizing responses. It concerns physical participation as well. This is in line with what Gill et al. (2021) have identified as qualitative obstacles to access to justice.

As one of the users said, courthouses are places of exclusion. And this is even more poignant when the buildings are not prepared to welcome people with disabilities. This is a matter of structural ableism (Lundberg and Chen, 2023).

*No one has thought, not even now, in the 21st century, about transforming the courts. Like many public departments, many public institutions, they forget there are people who have disabilities, and who have as many rights as those who move around easily*. [Litigant 4, 2011]

*There are no access ramps. Lots of stairs. It is not easy for an elderly person to go to that court. Climbing upstairs is not easy*. [Litigant 3, 2011]

Ableist architecture refers to the design of built environments that prioritize the needs of able-bodied individuals, often excluding or marginalizing elderly people or those with disabilities (American Bar Association, 1991; Allen, 2021). This can be manifested in numerous ways, from a lack of wheelchair ramps to the placement of essential services on upper floors that are only accessible via stairs. In this context, Lefebvre's lived space becomes a tool for critiquing ableist architecture. When we apply this to courthouses, we can see how the architecture of these spaces can reinforce ableist norms: a courthouse with a staircase leading up to the entrance presents a physical barrier to those who cannot navigate stairs, instead of symbolizing justice and authority. The irritation these users feel is precisely about that: a sense of missing respect, of injustice that derives from the fact that the same institution one seeks to claim rights is physically denying them. After all, courthouses are the pillars of our legal system, the guardians of our rights.

## 6 Concluding remarks

The courthouse, an emblem of the justice system, stands tall in the collective imagination. Its layout is meant to symbolize the promise of fair hearings and the legitimacy of the institution, depending on the viewpoint. However, reality often diverges from this idealized vision. Interviews reveal emotional turmoil.

In these spaces, like Nir and Musial (2020) claim, and interviews illustrate, emotions run high: frustration, fear, insignificance, loss, exclusion, distance, can be quite close to the surface and are also shaped by the physical design of the space (Dahlberg, 2009).

Based on the thematic analysis done and on the intersection with Lefebvre's concept of lived space, the following themes emerged from the different users' experiences:

- **Lived space as the inhabitants' experience of legitimacy lost:** the outdated facilities, and absence of proper conditions of the buildings, lead the professionals to speak of frustration and exhaustion, thus relating to a loss of symbolism, legitimacy, and authority of the judiciary and professionals.
- **Lived space as the inhabitants' experience of threat to objectivity and quality in decision-making and sentencing:** inhabitants' complaints about their working conditions reveal feelings of anger, insecurity, and powerlessness. These emotions impact their overall well-being and stress levels, ultimately influencing their decision-making processes.
- **Lived space as the inhabitants' experience of empathy:** acknowledging other peoples' emotional experiences inside the courthouse, particularly those of children, becomes crucial for ensuring just decision-making.
- **Lived space as the visitors' experiences of estrangement, distress, and rights' exclusion:** Distress, exposure, and irritation reveal a denial of justice, affecting people in unequal ways (children, the elderly, people with disabilities), emphasizes the need for more inclusive and supportive courthouse environments.

Court buildings are not just physical structures, they are made of conceived, perceived and lived spaces. They produce emotions, power dynamics and social relations. By considering the lived spaces of the diverse court users, architects and planners can begin to design courthouses that are more inclusive and accessible, challenging the exclusionary and distant assumptions often underpinned by the architectural design of courthouses, as the diverse narratives by visitors illustrated. Furthermore, and like inhabitants stressed, good working conditions are essential to promote decision-making processes. There is a need for a new approach to courthouse design, one that reflects the values of justice, fairness, respect, and also that of care, which should be at the heart of the (family) court system.

## Data Availability

The raw data supporting the conclusions of this article will be made available by the authors, without undue reservation.

## Footnotes

^1^The authors use Bens (2018) analysis of courtroom settings as affective atmospheres. The notion of the courtroom as an affective atmosphere was developed by Bens in an ethnographic study on the International Criminal Court (ICC) in The Hague. It describes the overall sensation that occurs at a specific time and space. Although this notion seems interesting, I will not deal with it for the purposes of this article as I engage with Lefebvre's dimension of the lived space, which extends beyond the courtroom, and directly connects to the individual emotions of different court users.

^2^The Chambre de la Jeunesse, or Youth Chamber, is one of the divisions of the Court of Quebec. It is competent to decide matters related to children at risk (until the age of 18 yo); adoption; child custody; parental responsibilities; and juvenile delinquency. For more information see: https://www.quebec.ca/justice-et-etat-civil/systeme-judiciaire/tribunaux-du-quebec/cour-du-quebec/chambre-de-la-jeunesse (last visited 12 August 2024).

^3^According to Faria (2018, p. 184), courthouse “user-inhabitants” refers to those working within court spaces, while court “user-visitors” encompasses those who visit said spaces for different reasons. Each group possesses distinct needs and expectations regarding the court buildings.

^4^Nordquist and Bergman Blix (2022) use the concept of emotional capital, building on Bourdieu's theoretical framework.

^5^Although generally identified with the tasks and powers exercised in the criminal field, the Public Prosecution Services have a polymorphic nature, which extends to the relevant functions assigned to it in other jurisdictional areas, among which the Family and Minors area. For more information see: https://en.ministeriopublico.pt/en/perguntas-frequentes/activity-areas (last visited 12 August 2024).

^6^I have decided to focus on litigants only, as user-visitors, because other visitors, such as legal counsel, have a more in-depth knowledge of the court spaces, even though they are not inhabitants. I wanted to concentrate on the litigants' point of view.

^7^Cf. http://www.cnnportugal.iol.pt/videos/falta-de-magistrados-profissionais-cansados-e-edificios-a-cairem-aos-bocados-um-retrato-dos-tribunais-nacionais/634d556e0cf2ea367d53a1ef (last visited 22 March 2024).

^8^A report based on an online survey carried out by the Judicial Institute of University College London and in which 99% of English and Welsh judges, circa 1600, took part. See Thomas (2017).

^9^On this question, see Danziger et al. (2011).

^10^Nordquist and Bergman Blix (2022) also interviewed a judge who reflected on the divergence between his own feelings of ease in the court setting and the anxiety felt by the people coming to court. This management of the feeling of ease in court, as well as being sensitive to the unease of others also relies, according to the authors, on the professional's emotional capital.

^11^Which resonates with what was mentioned by the judges and prosecutors.
